# A transdisciplinary framework for empathy research

**DOI:** 10.3389/fnhum.2026.1703147

**Published:** 2026-07-02

**Authors:** Kathryn K. Irish

**Affiliations:** 1 Department of Physiology, Neuroscience Program, Michigan State University, East Lansing, MI, United States; 2 School of Social Work, University of Michigan, Ann Arbor, MI, United States

**Keywords:** empathy, Edith Stein, theory of mind, theory of embodied simulation (TES), transdisciplinary, phenomenology, neural models

## Abstract

**Introduction:**

Empathy research spans the social, natural, and clinical sciences, yet its translational impact has been limited by conflicting conceptual frameworks and incompatible methodologies. This paper addresses these barriers by introducing a transdisciplinary framework that consolidates predominant theories of empathy and integrates contemporary neuroscience findings.

**Methods:**

A conceptual synthesis was conducted to reconcile major theoretical perspectives and relevant neuroscience findings from empathy research. Cross-disciplinary constructs, mechanisms, and methodologies were examined and organized into an integrative framework intended to support interdisciplinary translation and collaboration.

**Results:**

The resulting transdisciplinary framework unites Theory of Mind and Theory of Embodied Simulation by conceptualizing empathy as a process of social perception. Empathic experience is organized into four stages: (1) physiological mirroring, (2) affective resonance and responding, (3) cognitive understanding, and (4) appraisal/decision. Each stage is linked to its theoretical origins and associated with proposed neural mechanisms underlying self-other awareness.

**Discussion:**

By reframing empathy as a neutral process of social perception, rather than a subjective virtue or innate trait, this framework makes the construct more accessible to scientific inquiry. Conclusion: The proposed framework supports interdisciplinary synthesis and collaboration, thereby enhancing the translational potential of empathy research to inform clinical practice, civic engagement, and broader efforts to strengthen human connection.

## Introduction

The topic of empathy has been covered extensively for nearly two and a half millennia. As early as 6th century B.C., ideas resembling empathy were described in various Eastern philosophical traditions, most notably by Kong Qiu (孔子) (Latinized: Confucius) (Slote, 2010). Centuries later, early iterations of concepts related to empathy, such as intersubjectivity, arose within Western philosophy, in Aristotle’s 4th century writings pertaining to “*empathein,*” or the process by which humans “animate the inanimate” (Hunsdahl, 1967). During the 19th century German philosopher, Robert Vischer, wrote extensively about “*einfühlung*,” a conceptual predecessor to empathy, referring to a person’s ability to “feel into” another person or object (Bošnjaković and Radionov, 2018). In the 20th century, concepts pertaining to empathy formed the very field of psychotherapy, beginning with Freud (Bošnjaković and Radionov, 2018; Raines, 1990; Major and Miller, 1981; Kariagina, 2018), and later evolving into humanistic, client-centered interventions (Rogers, 1957).

Empathy is of central importance to the helping professions. Ideas about empathy are reflected in powerful social and civic institutions, and empathy itself characterizes the foundations of clinical practice (King and Holosko, 2012). Yet, there is a centuries-long debate concerning how to define and measure empathy. In fact, Stein (1917) aptly referred to this issue as, “Zum Problem der Einfühlung (On the Problem of Empathy)” in her seminal writing on the topic (Stein, 1917). Despite the acute need for conceptual clarity, there is no shortage of research on empathy. In fact, there have been over 70,000 scientific publications involving “empathy” in the past 25 years, alone (Scopus, 2025). Yet, it is likely that many of these studies simply replicate the same problems identified by Stein (1917), a century earlier, such as conceptual muddling, or assuming that empathy, in its entirety, reflects a simple mirroring process. These longstanding conceptual issues have led to modern problems, such that inconsistencies in operationalization of the empathy concept have limited the translational potential of cross-disciplinary findings. Despite significant time, effort and material resources, practical applications of empathy research, translated with the intention of addressing real-world problems—are difficult to find. Despite its foundational significance across all aspects of human life, and its vast representation in scientific literature, concrete contributions—such as interventions intending to improve diplomacy, or enhance social understanding, seem disproportionately low. This is a significant gap, as so many social problems (systemic and interpersonal, alike) are fundamentally connected to empathy.

Modern empathy research often involves interdisciplinary collaborations between cognitive and/or the social sciences and neuroscience, and most empirical literature on empathy can be described under two divergent theoretical camps: Theory of Mind (ToM) (Premack and Woodruff, 1978), which emphasizes cognitive aspects of empathy, including the ability to infer that other persons have their own first-person perspective, emotional experiences, thoughts and motives for behavior (Baron-Cohen, 2000); and the Theory of Embodied Simulation (TES) (Gallese, 2005) which emphasizes autonomic, non-conscious forms of mimicry and physiological simulation of experiences, which are interpreted as either facilitative predecessors of empathy, or are sometimes framed as “empathy” as a conceptual whole (Gallese, 2005; Gallese, 2019; Svensson et al., 2007). Each theory emphasizes entirely distinct dimensions, and because modern empathy research tends to fall under one camp or the other, when a study mentions “empathy,” there is remarkable variability in how empathy is conceptualized, operationalized, and measured.

This manuscript aims to reconcile and connect these theoretical camps, framing them as part of a larger sequence of social and emotional perception that is empathy. Framing the mechanisms of empathy as a sensory process, establishes a context for communication, and further elaboration downstream. But first, a shared framework and language must be established. Stein’s (1917) conceptualization of empathy offers a modern solution to this problem, as her framing of empathy as an experience “*sui generis*,” or “unique unto itself” captures dimensions from both Theory of Mind (ToM) and Theory of Embodied Simulation (TES) under a single paradigm.

This paper proposes a transdisciplinary framework for empathy research, rooted in the work of Stein (1917), that reconceptualizes empathy as a neutral, perceptual process. This paper deliberately reframes from examination beyond these fundamental perceptual mechanisms, as the central aim is to reconcile and organize these two empathy theoretical camps in a temporal model, framed concurrently with their proposed underlying neural mechanisms, much like how other sensory systems are presented in medical textbooks. Given that this model is simply a starting point, this paper aims to reconcile language and concepts reflecting only the perceptual mechanisms involved in empathy – without further exploration into behavioral outcomes, or other moral or social implications. Explorations of the behavioral, social and moral domain require a shared conceptual and lexical foundation, which as stated previously, is the barrier this paper intends to address. As such, this paper is neither comprehensive nor exhaustive in its conceptual analysis, rather, it is a frame to be built upon and expanding in accordance with new information, ideas and notions from the many theoretical camps not explored within the present work.

This framework integrates cognitive (Theory of Mind) and embodied (Theory of Simulation) accounts within a four-dimensional model including (1) physiological mirroring, (2) affective resonance and responding, (3) cognitive understanding, and (4) appraisal/decision, which are presented conjointly with their proposed neural substrates. The aim is to establish a cohesive lexicon, reduce conceptual fragmentation, and enhance the translational potential of empathy research across clinical, civic, and societal domains. As such, this model frames empathy as a perceptual process, in terms of a distinct sequence of neurophysiological processes, and a reconciliation of conceptual stages offered by Theory of Mind and Theory of Embodied Simulation, united under the empathy frame originally presented by Stein (1917). This approach aims to reduce conceptual ambiguity and improve translational consistency across disciplines by mapping theoretical constructs onto their proposed physiological correlates in a clearly defined, sequential framework, using existing evidence. This model isolates the perceptual mechanisms involved in empathy and organizes these in stages corresponding to their respective conceptual frames, thus establishing a unified lexicon that support a greater methodological consistency and conceptual clarity for future research on this topic.

### History of the problem

British psychologist, Edward Titchener first coined the term “empathy” early in the 20th century, as an English form of its conceptual predecessor “*einfühlung”* (Bošnjaković and Radionov, 2018; Hunsdahl, 1967). However, the definition of “empathy” has been debated across disciplines over the past century (Arancibia, 2021; Agosta, 2014; Bošnjaković and Radionov, 2018; Eisenberg and Miller, 1987; Hall and Schwartz, 2022; Hunsdahl, 1967; Kariagina, 2018; Raines, 1990; Stein, 1917). The notion of empathy as a kind of “*einfühlung”* represents a rare point of consensus amongst empathy theorists; at least, insofar as “empathy” is a word used to describe the process by which humans recognize and engage the distinct consciousness of other people (Bošnjaković and Radionov, 2018; Hunsdahl, 1967; Raines, 1990; Stein, 1917; Svenaeus, 2018), animals (Axelsson and Fawcett, 2020) and/or even a process through which humans attribute distinct consciousness to objects, such as art (Ardizzi et al., 2021) or aesthetic experiences (Taruffi et al., 2021). Beyond this, however, there is very little scholarly agreement.

Challenges related to achieving a consensus definition for subjective, broad-scope and innately relevant concepts, is not a problem unique to empathy; in fact, many transdisciplinary concepts such as “happiness” or “love” are similarly relevant to human life, and just as difficult to define philosophically and operationalize in research (Hall and Schwartz, 2022). Empathy, however, is unique in so far as it is deeply intertwined with morality and has been used to invoke many highly variable ideas concerning fundamental human “goodness.” From its earliest inception amongst Eastern religious traditions, circa 6th century B.C., discourse on subjects related to empathy have been inexorably tied to virtue and social morality (Slote, 2010). The connection between empathy and morality is highly relevant, as, throughout human history, there is a long and unsorted record of people taking ambiguously defined concepts, giving them a great deal of social power via the assignment of moral weight, and then erroneously employing these vague ideas to validate the provision of power amongst a “moral” few, and, likewise, to justify atrocities committed toward the “amoral” masses. Yet, just as there is potential to misuse the concept of empathy, there also exists a potential to mitigate this risk through conceptual clarification. Reframing empathy as a neutral, objective process of social perception may reduce potential misuse of this concept in social and civic realms. Furthermore, conceptual clarification may lead to greater potential for the reconciliation of cross-disciplinary research in a manner more conducive to translational application.

Decety and Jackson (2006) propose several relevant ideas in their model of empathy, including that (1) underlying neural architecture facilitates the emotional, affective, and cognitive awareness that characterize different aspects of empathy and (2) these aspects are not, individually or collectively, comprehensive representations of empathy, but rather its components. Their model frames empathy as an experience involving emotional sharing, self-other awareness (and distinction), and a cognitive understanding of the other’s perspective.

The ideas proposed by Decety and Jackson (2006) strongly align with the conceptual model proposed by Stein (1917) and with those presented here, which aim to reconcile the distinct elements comprising empathy as a perceptual process. However, unlike Decety and Jackson (2006), whose work often employed indirect indicators of activity (i.e., fMRI), the present paper focuses on more on direct physiological measures of brain activity; moreover, there is no attempt to further extrapolate or interpret the interpersonal implications of this activity beyond their respective construct(s). This is an important distinction that orients the scope of this paper, differentiating it from similar works which attempt to understand neural activity in terms of behavioral or interpersonal outcomes. Rather, the central aim here, is to refine a neutral perceptual model of empathy as a function of both physiological mechanisms (e.g., affective blindsight, mirroring) and internal affective and cognitive processes (both conscious and nonconscious) in order to present a unified model for the mechanistic aspects of empathy, inclusive of clearly defined conceptual domains and respective tools of measurement, with the goal of mitigating methodological inconsistencies and increasing translational potential.

### Empathy as a philosophical concept

German philosopher, Edmund Husserl (1859–1938) wrote extensively on the topic of intersubjectivity, or the mutual awareness and shared understanding which arises between people during social interaction. Husserl’s work is considered the seminal foundation of phenomenology, a branch of philosophical thought primarily interested in understanding the nature of the first-person, conscious experience (Smith, 2018). Phenomenology forms a kind of epistemological bridge between positivism and interpretivism. Positivism assumes a finite, objective, universal reality, while interpretivism emphasizes the social construction of reality and expresses explicit interest in interpreting meaning and relations between groups, often examining differences in power with specific aims of critique. Phenomenological inquiry often frames explorations using empirical tools, which imply the notion of a finite, measurable reality, however, phenomenology is fundamentally interested in understanding experiences at the individual level, that is, how people perceive the world – a process that overtly rejects the positivistic notion of a universal reality, and instead explicitly focuses on inherently subjective nature of reality (Smith, 2018). As such, a phenomenological approach is exploratory, experience-focused, and subjective, making it well-suited to explore concepts such as empathy.

A significant portion of philosophical work has addressed the epistemological roots of empathy and both its distinction from, relatedness to, various phenomena including emotional sharing (Zahavi and Rochat, 2015), sympathy, compassion, altruism (King and Holosko, 2012), imitation, emotional contagion, *mitfühlen* (feeling “for” someone), *einsfühlen* (feeling “one with”), *einfühlung* (to recognize or project feeling in another), *miteinanderfühlen* (feeling in unison, as a pair or group), etc. (Szanto and Moran, 2020; Stein, 1917). These distinctions are acknowledged as relevant to the broader understanding of intersubjective processes but are outside the scope of the present paper, which instead focuses on distinguishing and clearly capturing the distinct mechanisms that comprise the neutral perceptual processing that characterize empathy. The central notion of this paper is similar to that which is described in the Decety and Jackson (2006) model, and echoed by Agosta (2014), which frame empathy as a facilitatory mechanism underlying interpersonal interaction. The present paper extends this view, further yet, framing empathy with even greater neutrality, as a distinctly staged perceptual process, whilst also presenting a unified framework and tool that can be easily employed by cross-disciplinary researchers, in order to mitigate methodological inconsistencies, and increase the translational potential of findings from empathy research.

### Empathy as a clinical concept

Empathy became a clinical concept in the early 20th century with the emergence of psychoanalysis and the writings of Freud, who characterized it as an exchange of mental and emotional content across a liminal space, emphasizing its curative potential (Bošnjaković and Radionov, 2018; Major and Miller, 1981). Empathy is a requisite aspect of the therapeutic process, and a tool used in humanistic, client-centered therapy, where the objective is to help clients accept themselves, change as they see fit, and live their most authentic life (Rogers, 1957). As a clinical concept, empathy is described as a unique form of being present and listening associated with “clinical intuition” (Rogers, 1986; Fox et al., 2016). Similar to its philosophical predecessors, clinical empathy involves both an urgent awareness and a deliberate decision to relinquish one’s own perspective (i.e., a temporary dissolution of the “self-other” boundary), characterized by immersion in another space, perspective, or mindset, resulting in improved understanding and subsequent ability to assist or support (Freud, 1912; Major and Miller, 1981; Stein, 1917; Rogers, 1986).

There are clear parallels between Freud (1912), Stein (1917), and Rogers’ (1986) descriptions of empathy and clinical intuition in that both involve an urgent awareness and a deliberate decision to relinquish one’s own perspective, a process described by Rogers (1957) as “a willful suspension of the self” and by Stein (1917) as a rather concrete decision. Both depictions involve some alteration of perceptual or “conscious” awareness and are followed by a temporary immersion in another space, perspective, or mindset. Freud, Stein and Rogers all appear to be describing their experiences of “clinical intuition” and/or “empathy” in terms of immediate awareness, followed by immersion in a mental and emotional space, characterized by sensory experiences arising from various perspectives, and ultimately concluding with some improved understanding and ability to assist, support or help (Freud, 1912; Stein, 1917; Rogers, 1986). These somewhat fantastical descriptions of clinical intuition as a form of empathic understanding involve an element of paradox – they are both immediate, urgent, and non-conscious, but also deliberate, intentional and goal-directed. They characteristically involve a relinquishing of one’s first-person vantage point, in exchange for “altered consciousness” as Rogers’ (1986) describes – yet, the clinician maintains full awareness, and is often able to experience themselves, while simultaneously assuming the perspective of another, and can differentiate the two perspectives clearly. Maintaining a clear self-other distinction is another critical domain of empathy as a state of mind (Little et al., 2022). It remains critical to emphasize that the accuracy of information derived through empathy or clinical intuition is unclear, and the process itself is not immune to interference from the clinician’s internal circumstances. Rogers’ (1957) dedicates special attention to acknowledging this interference transparently, and encourages clinicians to consciously, willfully maintain a vigilant self-awareness to attempt to diffuse or mitigate such “intrusions of self.” Descriptions of empathy as both a clinical, and a philosophical concept, support the notion of empathy being more of a neutral, non-valanced process of perception, involving a sequence of relatively distinct phases – alternating between varying degrees of “self-other” awareness. In other words, empathy, in both philosophical thought and clinical application, involves both intentional and unintentional social awareness, with a level of concurrent effort toward making sense and responding to this information – much like any other form of sensory perception. The difference with empathy is that the focus shifts between perceiving oneself, and perceptions of another.

There is no subsequent implication or judgement necessary for empathy; no decisive altruistic act, or even intention to help (outside clinical contexts)– empathy is fundamentally a neutral process of observing and attempting to know and understand another; any subsequent level of intention beyond this neutral awareness may be better characterized by other concepts such as compassion, curiosity or even malintent. Likewise, the behavioral domain is relevant within this conceptual lineage, as it represents the cumulative outcome of perceptual processes, and are crucial for understanding empathy’s role in interpersonal functioning. However, similar to intention, or any other cumulative outcome of a perceptual process, the behavioral domain is beyond the scope of the current model. Future work may expand upon this framework to incorporate behavioral outcomes and related psychological processes. For now, the focus is limited to reconciling theoretical foundations, conceptual and operational definitions, and neurobiological mechanisms underlying the perceptual processes described as empathy.

Framing empathy as a perceptual process is especially relevant in defining the present aims, as perceptual processes are mechanistically oriented, and un-valanced. Although the manner in which sensory information is received, organized and interpreted is very much vulnerable to bias (Friston and Kiebel, 2009), this paper focuses on grouping the cellular and molecular activities which constitute these sensory processes, which are generally consistent among mammals. In other words, this paper is focused on classification, matching and aligning measures and constructs with their very fundamental neural underpinnings, and is deliberately abstaining from further exploration of downstream activities, such as behaviors, decisions or intention(s) that arise as a result of moving through this perceptual process.

Several domains of modern empathy research are interested in the philosophical and/or interpersonal arms of empathy in term of childhood development, pathologies and/or prosocial behavior (Decety and Holvoet, 2021). In contrast, the aims of this present work are primarily functional: (1) to synthesize some (but not all) of the most employed empathy theories to define distinct categories relating to the perceptual process of empathy (2) to reconcile the tools and operationalizations commonly employed in contemporary research to assess each domain and (3) offer a shared lexicon and reliable framework to facilitate interdisciplinary research, reduce methodological inconsistencies in both concept and measurement across social and natural sciences, and ultimately increase the translational potential of findings from within this body of research.

### Reconciling modern theories of empathy

In this section, two primary empathy frameworks will be discussed: Theory of Embodied Simulation (TES) (Gallese, 2005) and Theory of Mind (ToM) (Premack and Woodruff, 1978). Please note, Theory of Embodied Simulation (TES) is distinct from the Theory of Empathic Sensitivity (TeS), although the acronyms are similar. Whether or not explicitly stated within a given study, it seems the majority of social science and neuroscience research seems to be oriented under one or both of these theoretical orientations; explicitly, by invoking the theory directly, or implicitly, by the framing and methodologies used to study empathy, as either a process of non-conscious mirroring (i.e., TES) or cognitive deliberation (i.e., ToM).

### Theory of embodied simulation (TES)

The Theory of Embodied Simulation (TES) emphasizes the role of physical and emotional mimicry and simulation as a means of understanding the experience of another person (Gallese, 2005; Gallese, 2019; Svensson et al., 2007). TES is often described in conjunction with “mirror neurons” which offer a mechanism for these experiences and/or emotional contagion effects (Cattaneo and Rizzolatti, 2009; Kilner et al., 2009). TES describes empathy as a series of autonomic, non-conscious forms of mimicry and physiological simulation of experiences (Gallese, 2005; Gallese, 2019; Svensson et al., 2007).

It is not uncommon for modern studies to fail to clarify how the role of mirroring in empathy is framed in the study design; rather a vast majority of otherwise rigorous studies simply measure physiological mirroring and then report findings of mirroring under a not-otherwise-specified variable of “empathy;” thus, suggesting via overt statement or implication that imitation or mirroring, alone, is wholly representative of “empathy” (Anderson et al., 2021; Ardizzi et al., 2021; Taruffi et al., 2021; Birch-Hurst et al., 2022; Galang et al., 2021; Genzer et al., 2022; Harjunen et al., 2021; Jie et al., 2021; Schmidt et al., 2021; Wang et al., 2021; Yu et al., 2022; Axelsson and Fawcett, 2020). Other studies invoking a TES frame may more cautiously infer mirroring as facilitative, predecessors of empathy (Albrecht and Bellebaum, 2021; Simon and Gutsell, 2021).

### Theory of mind (ToM)

Theory of Mind (ToM) (Premack and Woodruff, 1978) describes a person’s capacity to attribute mental states to both oneself, and to others (Baron-Cohen, 2000; Premack and Woodruff, 1978). In this framework, empathy is a special form of social cognitive processing that involves recognition of one’s own and other person’s distinct first-person perspectives (Baron-Cohen, 2000; Premack and Woodruff, 1978). Theory of Mind frames empathy in terms of information-processing, based on inferences from one’s own experience, and heavily emphasizes cognitive aspects of social perception, such as attention, memory and reasoning reliant on existing mental representations (Baron-Cohen, 2000; Premack and Woodruff, 1978).

### Empathy: trait, state or an experience “*sui generis*”?

In research, empathy is often assessed as a trait (i.e., a static, unchanging aspect of one’s personality) (He et al., 2022; Little et al., 2022); a state (i.e., a distinct, but transient response to a specific event) (Ardizzi et al., 2021; Birch-Hurst et al., 2022; Blagrove et al., 2021; Genzer et al., 2022; Harjunen et al., 2021; Jie et al., 2021; Rodrigues Tavares et al., 2021; Schmidt et al., 2021; Simon and Gutsell, 2021; Yu et al., 2022) or a combination of both a trait and a state (Albrecht and Bellebaum, 2021; Anderson et al., 2021; Axelsson and Fawcett, 2020; Galang et al., 2021; Taruffi et al., 2021; Wang et al., 2021; Zhao et al., 2022).

Trait empathy is typically assessed using standardized, validated self-report measures such as the Interpersonal Reactivity Index (IRI) (Davis, 1983), the Toronto Empathy Questionnaire (TEQ) (Spreng et al., 2009), or the Cambridge Behavior Scale – Empathy Quotient (Baron-Cohen and Wheelwright, 2004). These measures are multi-dimensional and commonly address affective resonance, (ex., TEQ: *When someone is feeling excited, I feel excited, too*); cognitive understanding (ex., IRI: “*I try to look at everybody’s side of a disagreement before I make a decision*) and deliberative aspects of cognitive or affective responding (ex., TEQ: *When I see someone being taken advantage of, I feel protective towards them*). Assessing empathy as a trait comes with ethical problems, as “traits” are largely considered non-changeable aspects of one’s personality, and as previously discussed, empathy as a concept, heavily invokes social morality.

When empathy is discussed as a “trait” it is often applied to vulnerable or incarcerated populations, or others living on the social margins, who are often assumed to have “less empathy” than the typical person (Fallon, 2013; He et al., 2022). In such studies, researchers may uncritically attempt to explain complex social behaviors in terms of biological factors, such as anatomical or functional differences in brain activities (i.e., Fallon, 2013) or genetic polymorphisms, which are described in association with varying levels of “morality” (i.e., He et al., 2022). This frame, though well intended, may be interpreted by some as a new kind of biological determinism, which holds potential to be incredibly damaging.

However, most social science and neuroscience collaborative research on empathy, involves measuring empathy as a state (Ardizzi et al., 2021; Birch-Hurst et al., 2022; Blagrove et al., 2021; Genzer et al., 2022; Harjunen et al., 2021; Jie et al., 2021; Rodrigues Tavares et al., 2021; Schmidt et al., 2021; Simon and Gutsell, 2021; Yu et al., 2022). While standardized self-report measures to assess state empathy exist, such as the State Empathy Scale (SES) (Shen, 2010) and continue to be used in modern empathy research (Blagrove et al., 2021), there are many other neurophysiological and imaging techniques that are more commonly used. In modern research, physiological mirroring is generally measured using either direct electrophysiological measures of brain activity, such as electroencephalogram (EEG) (Albrecht and Bellebaum, 2021; Birch-Hurst et al., 2022; Galang et al., 2021; Genzer et al., 2022; Harjunen et al., 2021; Schmidt et al., 2021; Simon and Gutsell, 2021; Wang et al., 2021; Yu et al., 2022) or indirect measures such as functional magnetic resonance imaging (fMRI) (Anderson et al., 2021; Ardizzi et al., 2021; Jie et al., 2021; Taruffi et al., 2021; Zhao et al., 2022).

These tools are employed to assess a physiological and/or affective response to a social circumstance. Unlike trait empathy, there is an assumption that state empathy is a time-limited occurrence, characterized by varying levels of intensity and duration. This definition is perhaps more useful than trait empathy but also has limitations; specifically, there is heavy reliance in the assumed connection between non-conscious, involuntary, physiological responses and the subsequent extent to which they influence deliberative actions or manifest in other complex examples of social responsiveness.

These two frameworks – trait, or state, are the foundation of the many conceptual and methodological differences which prevent scholarly synthesis of empathy research and inhibit translational potential. In fact, it is not uncommon for studies to refer to the construct of “empathy” without any further explanation, leaving the reader to infer any further connotation from measures employed to assess said construct. This has resulted in the mountain of unsorted socks that is modern empathy research – a great mass of irreconcilable operationalizations all claiming to assess “empathy.” By establishing a set of criteria for evaluating and defining different domains of empathy, broadly framed as a perceptual process, perhaps a foundational lexicon can begin to take shape.

### Empathy has no valence, and it is not a behavior

Despite its conflation with moral virtue and positively valanced concepts such as compassion and/or altruism; at its philosophical core, there is no requisite connection between empathy framed as a perceptual process, and prosocial behavior (Eisenberg and Miller, 1987). Consider, for example other sensory processes, such as hearing or seeing. One may hear or see many different things, good or bad, but the process of seeing or hearing itself is not inherently an experience that is positive or negative. In the context of empathy, framed similarly as a sensory process involving perception of social information, there is no requisite type of behavior in response to what is perceived that makes the process of perceiving “empathy” as opposed to something else. In fact, one may accurately infer and understand the experience of another, move through all the typical stages of understanding that characterize “empathy,” and instead of deciding to respond in a prosocial manner, they may choose to not respond at all, or even to respond in a manner that further exacerbates the harm. The process of taking in and making sense of this information remains the same, a neutral process of perceiving and interpreting external information from other people, within a given social context.

Why, then, is the concept of empathy so often conflated with other positive social responses? This may be a social or cultural facet, or it may be the result of empathy being a response tied to some extent to oneself, or how one views, understands and frames themselves in a given circumstance; it could simply be that most people view themselves as compassionate or altruistic, as within the greater cultural context, these characteristics are valued. Therefore, this assumption about oneself, is extrapolated to others as well, leading people to the implicit assumption that others, like them, have prosocial intent and react in accordance in response to social perceptions. Perhaps this is partially responsible for the conflation between empathy and prosocial intent. However, just as understanding another person can be used to meet prosocial ends, like comfort or support; this same understanding can be employed to manipulate, exploit or harm. The process of empathy is simply a means through which one employes their faculties, intentionally and unintentionally, to recognize, take in/process, and understand social, emotional, physical and contextual experiences, occurring outside oneself. What one does with this information reflects the individual and their orientation to the greater social circumstance; it is not of the presence, absence, abundance or shortage of empathy.

### Empathy as a process

Edith Stein (1891–1942) studied under Edmund Husserl, the architect of phenomenology at the University of Freiberg in Germany. Stein completed her doctorate summa cum laude in 1917 with her thesis, *Zum Problem der Einfühlung (On the Problem of Empathy)* (Pontifical Council for the Liturgy, 1998; Szanto and Moran, 2020). Despite her academic achievements, she was repeatedly denied habilitation because of her gender and Jewish heritage (Szanto and Moran, 2020; Pontifical Council for the Liturgy, 1998). In 1922, Stein converted to Catholicism; her conversion strained family relationships and shaped her writings on faith, humanity, and empathy (Szanto and Moran, 2020; Pontifical Council for the Liturgy, 1998). In 1933, Stein was dismissed from her academic appointment under Aryan Laws, and in 1934, she entered a Carmelite convent. During this time, Stein actively sought to re-humanize Jewish families during Nazi persecution, even petitioning the Pope to condemn Hitler, though her appeals were ignored (Pontifical Council for the Liturgy, 1998). Arrested by the Gestapo in 1942, she and her sister were deported to Auschwitz and executed (Pontifical Council for the Liturgy, 1998). Later canonized as the only Jewish Catholic saint, Stein’s intertwined identities and historical context informed her view of empathy as a neutral perceptual process that, when intentionally applied, could counter dehumanization and foster social connection (Szanto and Moran, 2020; Pontifical Council for the Liturgy, 1998).

Stein’s (1917) dissertation *Zum Problem der Einfühlung (On the Problem of Empathy)* when viewed in the context of her life, makes clear the connections between empathy and (de)humanization, and the potential role of empathy in mitigating some of the most severe social atrocities. Stein (1917) asserted that the “problem of empathy” is fundamentally a problem of definition, noting an abundance of “mingling” concepts which clouded further philosophical and scientific exploration, Stein described a need to differentiate “empathy” from its conceptual kin and to establish a “requisite foundation” for any further presentation of ideas, discussion, or critique on the subject (Stein, 1917, pp.1). Stein (1917) does just this, conscientiously working through the epistemological foundations of the concept, ultimately offering a consolidated frame from which empathy can be understood as an experience “*sui generis*” (unto itself), involving a series of sequenced events: (1) emergence of the experience; (2) the fulfilling explanation; and (3) the comprehensive objectification of the explained experience (Arancibia, 2021).

#### Emergence of the experience

The “emergence of the experience” is described as a “sudden awareness” or apparition; an immediate knowing or recognition of the affective state of another person without requiring any conscious effort (Stein, 1917). Stein was critical of ideas that framed empathy as pure imitation (mimicking or simulating another), associative (relating to another by way of one’s own experience) or as a simple abstraction of one’s own experience, in analogical relation to another (Arancibia, 2021; Szanto and Moran, 2020). In acknowledgement of the conceptual entanglement between imitation and experience, Stein characterizes the relationship as follows, “Experience and expression are so closely associated that when one occurs, it pulls the other after it” (Stein, 1917, pp.24). The possibility of physiological replication and/or transmission of emotions via contagion effects is also acknowledged (Stein, 1917). However, Stein was careful to differentiate empathy from mirroring, simulation, or contagion effects; instead, framing imitation as a vehicle to gaining understanding (Stein, 1917). The role of physiological embodiment is more or less addressed as an aspect, predecessor and/or facilitator of empathy, but one that is fundamentally distinct from the holistic experience of empathy, *sui generis* (Szanto and Moran, 2020; Stein, 1917).

#### Fulfilling explanation

The second phase of Stein’s (1917) empathy involves a conscious decision concerning what to do with this sudden awareness of the other. Should the empathizer make an intentional decision to “inquire into the implied tendencies” (pp.10) of the empathee, and “bring the mood of the other into clear givenness,” (pp.10) in effort to achieve a level of understanding, then they can expect to be “pulled into” (pp.10) or immersed in the original experience, moving through it in such a way where one can view the object (empathee) and their relation to the experience in a unique way (Stein, 1917). In terms of intersubjectivity, this process is clearly described in terms of self-other distinction, whereby the subject (empathizer) views the object (empathee) separately, but from an original, immersive perspective.

Similar to many other conceptualizations of empathy as a dynamic, sequential experience (Freud, 1912; Rogers, 1957), Stein (1917) also describes the process of understanding as immersion, described in terms of relocation, or being “pulled into” (pp.10) an experience. Interestingly, many empathy theorists have described this immersive “viewing” process in terms of spatial location, as well, describing a process of being “moved or moving” (Freud, 1912) or “taken and immersed” (Rogers, 1957) into the space where empathy occurs. This experience has also been framed in terms of mental state, such as imagination or fantasy (Davis, 1983); a distinct plane of liminal consciousness (Freud, 1912) or, broadly, in terms of “an altered state of awareness” (Rogers, 1986). Stein (1917) describes the process of empathy in terms of the soul, whereby understanding occurs as the soul shifts perspective, as opposed to one’s mental state or consciousness.

#### Synthesis of experience

The final step in Stein’s (1917) empathy process is indicated by having achieved a “fulfilled clarification of the experience” (pp.10), at which point the subject (empathizer) may return to oneself with this new bit of knowledge and understanding of the other. From here, the empathizer may integrate this understanding into their perspective, where it may lead to downstream behaviors, thoughts or feelings. In this sense, Stein (1917) is framing empathy as a neutral, sequential process; a means of recognizing, taking in or perceiving and ultimately gaining a level of understanding regarding the social, emotional, physical and spiritual experiences occurring beyond oneself, had by other people in one’s immediate environment. This conceptualization of empathy differs remarkably from its conceptual successors, which seem to focus solely on the process of recognition and/or understanding, framing each as representing the entirety of the concept of “empathy,” if not overtly, then by omission of these other resonant elements.

The multiple levels of *sui generis* experience described by Stein (1917) uniquely capture empathy is a conceptually distinct, multi-dimensional process occurring simultaneously across many different levels of awareness and experience. Most modern studies operationalize only a handful of these aspects of experience, and this is one of the ways the conceptualization problem has resulted in limited translational potential of findings of empathy research. Interestingly, both Stein’s conceptualization of empathy, and the ideas she critiques as incomplete are very much the same that exist today, under the primary theories that characterize empathy in modern discourse: Theory of Mind (ToM) (Premack and Woodruff, 1978) and Theory of Embodied Simulation (TES) (Gallese, 2005). The stages Stein (1917) describes align neatly with the dimensions of ToM and TES, however, unlike Stein’s conceptualization, too often, when used in research, these theories are often considered mutually exclusive. Broadly, studies employing primary neuroscience techniques tend to fall under TES, measuring empathy as a state of mind indicated by physiological mirroring and affective resonance, while studies measuring empathy under a ToM paradigm focus more on cognitive aspects and/or the impact of one’s response on subsequent mood or behavior.

### A transdisciplinary model of empathy

**Figure 1:**
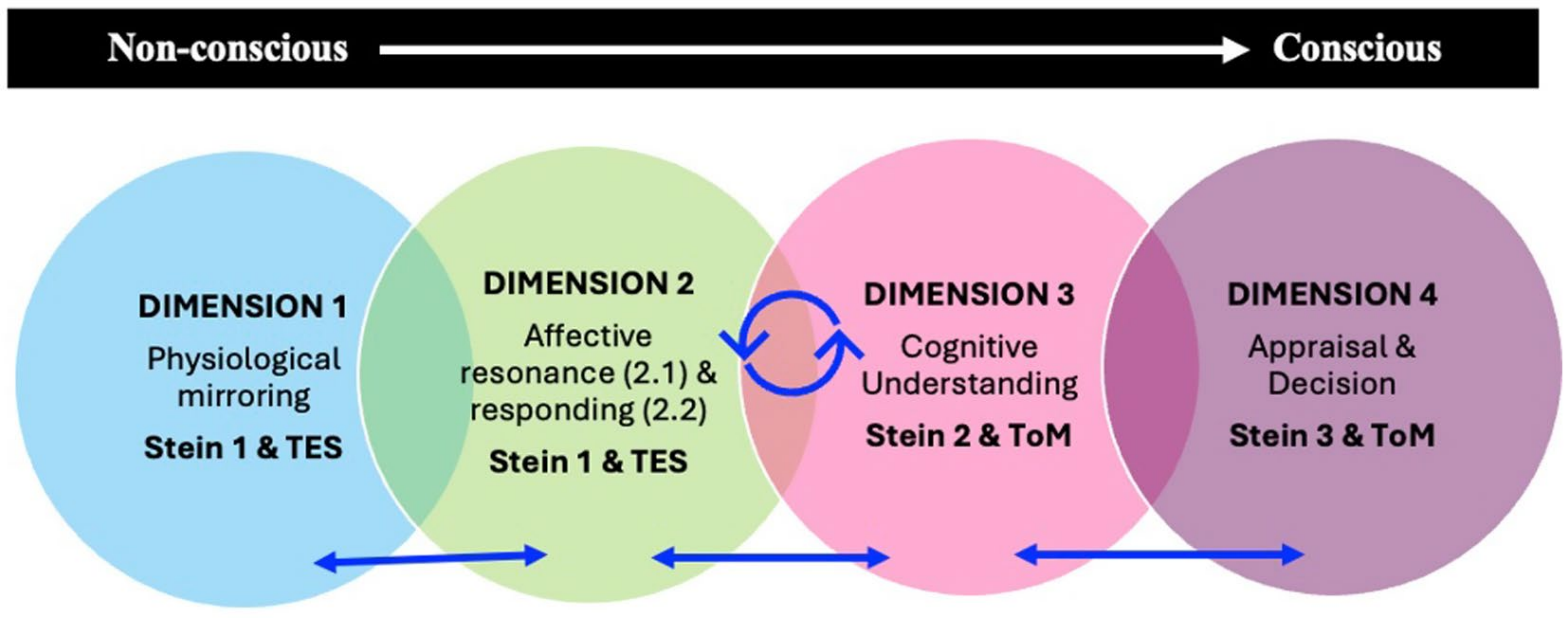
Four stages of empathy as a perceptual process. Integrates Stein (1917), Theory of Embodied Simulation (TES) and Theory of Mind (ToM) across four stages representing increasing levels of conscious awareness: (1) physiological mirroring, (2) affective resonance and responding, (3) cognitive understanding, and (4) appraisal/decision. Arrows indicate dynamic interaction between dimensions.

**Figure 2:**
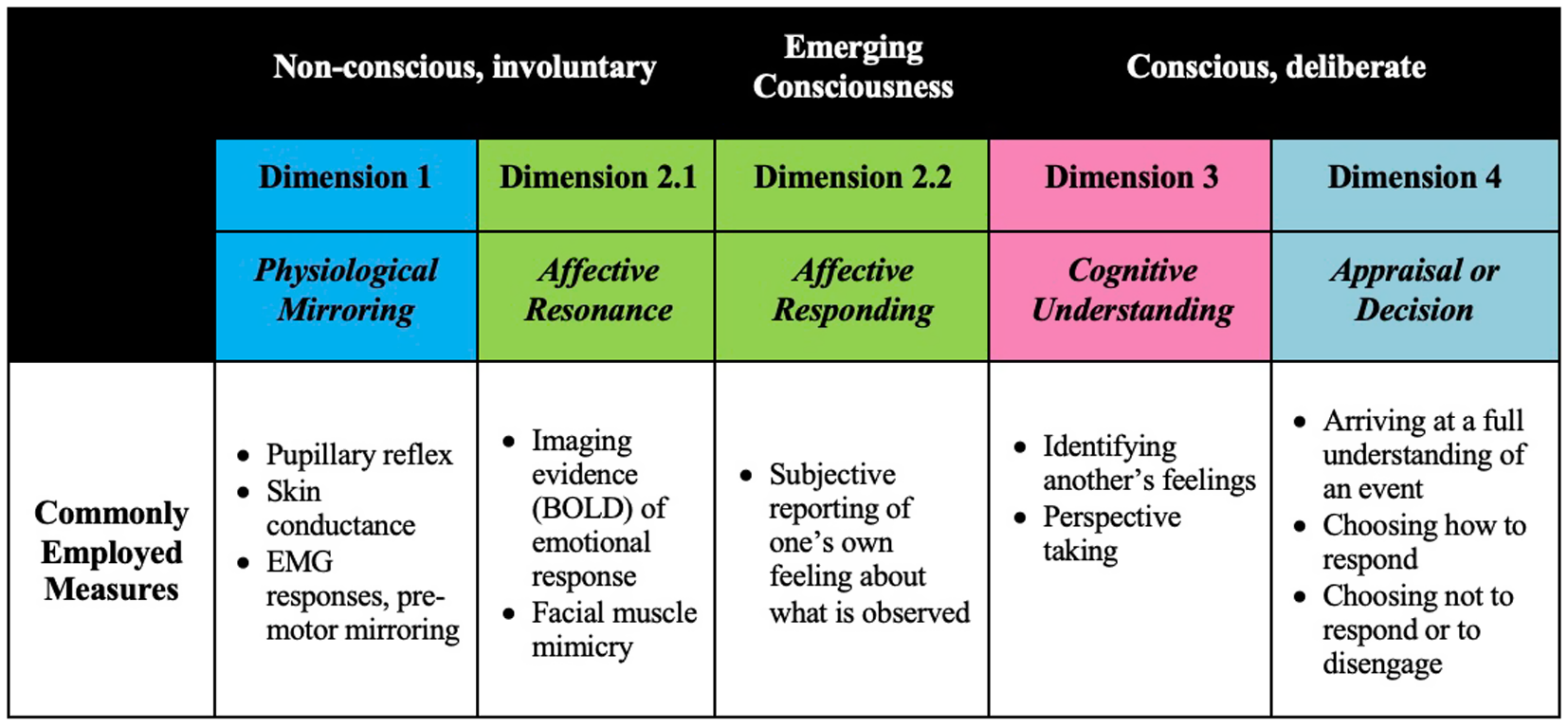
Empathy measures organized within transdisciplinary framework. Common research methods are grouped according to the four proposed dimensions of empathy, illustrating how existing assessment tools map onto distinct perceptual processes.

Below, a framework based on Stein’s (1917) comprehensive description of empathy is presented. This framework identifies four dimensions of empathy: (1) physiological mirroring (2) affective (2.1 affective resonance and 2.2 affective responding) (3) cognitive understanding and (4) appraisal/decision. Stages are distinct and consecutive but are proposed here as non-linear; for example, a person may receive and process both affective and cognitive information, concurrently, or circle back through the same domain multiple times, before steadily proceeding. The aim of this section is to define empathy as a neutral perceptual process, with distinct stages, under a unified transdisciplinary model, in order to establish a shared lexicon for cross-disciplinary synthesis, translation and application of findings in ways that may contribute tangible solutions to social problems. An abbreviated model is depicting theoretical integration into this model is shown in Figure 1, while Figure 2 depicts common measures associated with each domain.

#### Dimension 1: physiological mirroring (involuntary; self-directed)

The phenomena described under dimension 1 are consistent with those described within the Theory of Embodied Simulation (TES) and within Stein’s (1917) first event, the “emergence of experience” in the greater temporal sequence of empathy processing. Specifically, activities in dimension 1 are non-conscious, involuntary and reflect what is observed (i.e., mirroring) in an automatic manner. This dimension refers to the involuntary, automatic somatosensory emulation of observed movement, carried out by a network of neurons referred to colloquially as “mirror neurons” (Cattaneo and Rizzolatti, 2009). Somatosensory mirroring refers to the phenomena whereby areas of the brain associated with volitional movement respond to movements or sensations observed in others, as if the brain was experiencing them firsthand (Cattaneo and Rizzolatti, 2009). Evidence from event-related potential studies (ERP) suggests physiological mirroring phenomena (also described as “mimicry”) described in dimension 1 are sequenced and faciliatory, precipitating later affective stages of empathy perceptual processes (Sessa et al., 2018; Meconi et al., 2018). However, the influence of mirroring processes in dimension 1 may differ as a function of other facts, as some evidence suggests that the impact of physiological mirroring (i.e., “mimicry”) may be of greater relevance to persons with higher empathy scores as assessed via the Empathy Quotient (EQ) (Sessa et al., 2018). As such, phenomena captured here are framed as sequential, but the impact of dimension 1 may be best understood as a gradient – rather than a universally influential or requisite aspect of empathy perceptions.

#### Dimension 2: affective resonance and affective response (self-directed)

Dimension 2 comprises two components. The first is affective resonance, defined here as a nonconscious mirroring response in which a person briefly experiences the emotion observed in another. This process begins automatically and gradually becomes conscious toward the end of Dimension 2, marking a transition into Dimension 3 (the cognitive stage). In other words, an individual may first feel an emotion and recognize it as their own before realizing that it originated from another person’s experience. The second component, affective responding, follows this realization, representing both the original emotional experience, awareness of it originating from outside oneself, and further evolving into one’s own individual’s unique emotional reaction to what has been perceived. The sequence begins as non-conscious mirroring, evolves into an mirrored emotional response that at first, which is gradually recognized as mirroring. At this point, a new emotional response begins, originating not from an external observation, but from awareness of one’s own emotional reaction to having observed another emotional reaction in a separate individual.

##### Dimension 2.1: affective resonance

Events in dimension 2.1 are similar to those in dimension 1, in that both are automatic, non-conscious and involuntary; only, while dimension 1 characterizes a physiological response (e.g., pupillary dilation or skin conductance, etc.), dimension 2.1, refers to the innate, immediate, non-conscious mirroring of the emotional responses observed. A case could be made that affective resonance is evidenced by physiological responses such as pupillary dilatation (Axelsson and Fawcett, 2020; Decety and Holvoet, 2021); and it may be that later iterations of this frame will merge dimension 1 with dimension 2.1. However, at this point, it seems worthwhile to discuss each individually, as physiological responses include other, non-affective forms of mirroring, whereas events in dimension 2.1 exclusively refer to responses that mirror an observed emotion. As such, there is a minimal threshold between dimension 1 (physiological mirroring) and 2.1 (affective resonance), and the two share conceptual overlap, and likely occur concurrently. For example, when watching a scary movie, for a brief instant, a person may experience a fleeting emotion that is simply mirroring what is observed on the screen (dimension 2.1), before that feeling evolves into its own affective response (dimension 2.2), usually involving some recognition that the experience is being observed, and not actually occurring.

The non-conscious affective resonance in dimension 2.1 is often framed in terms of social perceptions and evolutionary advantage (Bonini et al., 2022). Broadly, affective resonance is often described in terms of a secondary mirroring system (limbic mirroring) which involves the insula and anterior mesial frontal cortex and recognizes and simulates affective responses in others (Cattaneo and Rizzolatti, 2009). Limbic mirroring, similar to physiological mirroring, is also involuntary, and non-conscious, and is typically associated with facial mimicry (Enticott et al., 2008). Limbic mirroring is also described affective resonance and is considered to play a role in emotion recognition (Enticott et al., 2008). Studies aimed at measuring this arm of the affective domain of empathy often do so using physiological assessment tools, or brain imaging to infer affective responses based on indirect evidence (Birch-Hurst et al., 2022; Galang et al., 2021; Wang et al., 2021).

##### Dimension 2.2: affective response

Affective responding (2.2) refers to the process whereby a person moves beyond autonomic responses, mirroring what has been observed, and begins to develop their own emotional reaction to the circumstance. This aspect typically involves a level of distinction between what is being observed, versus what is happening to the observer. Both dimension 1 (physiological mirroring) and dimension 2.1 (affective resonance) are mirroring processes, whereas dimension 2.2 (affective response) requires a distinction between self and other, which involves emerging conscious awareness, and some cognitive processing as well. Instead of mirroring a response occurring in another person (e.g., affective resonance), affective responding (2.2) originates from within the individual observer. The process of transitioning from affective resonance (2.1) to affective response (2.2) may be characterized by a kind of cycling through a level of cognitive processing, whereby there is an emerging awareness of this distinction, and the individual begins to form their own, novel emotional response. In this sense, dimension 2 captures elements of both Theory of Mind and Theory of Embodied Simulation and is similarly characterized as a transition point by Stein (1917) in her first stage of empathy, “emergence of experience” whereby the observer, having been “struck by sudden awareness” transitions back into their own vantage point, and begins to process what they are observing.

#### Dimension 3: cognitive (other-focused)

Dimension 3 is commonly described as “cognitive empathy” and is largely captured by Theory of Mind, and Stein’s (1917) stage of “fulfilling explanation”. It begins with a clear “self-other” distinction that marks an end to the involuntary responses to observed movements and affective states (Cattaneo and Rizzolatti, 2009) which are described in this model under dimensions 1 and 2. The initial, mirroring responses are recognized as foreign and are differentiated from one’s internal emotional response. It is this self-other distinction also is what differentiates “empathy” from related concepts such as sympathy, (feeling with someone) or compassion (feeling toward someone, motivated to help or respond) (Szanto and Moran, 2020) which are related, but different for two reasons (1) these concepts imply a specific prosocial intent and (2) they lack the requisite self-awareness that uniquely characterizes empathy. In other words, empathy infers a level of acute self-awareness concerning the boundary between self and other; stepping outside of it requires intention and a deliberate effort to understand an experience from a new perspective, through a process described by Carl Rogers as a “willful suspension of self” (Rogers, 1957). While Rogers (1957) is referring specifically to clinical empathy, which does have the explicit intention of understanding to help another person, the concept of stepping beyond oneself is unique to empathy and is similarly described by Stein (1917) as a conscious decision to attempt to understand.

As dimension 3 evolves, a person can begin to interact intentionally with what is being observed, reconciling observations with other environmental, contextual and/or social cues, one’s own memories and/or even one’s thoughts or values as they may relate to the observation.

Essentially, the cognitive domain of empathy refers to the ability to accurately recognize the emotional state of another person, which is sometimes described as “empathic accuracy” (Simon and Gutsell, 2021; Genzer et al., 2022); and to understand the experience from a perspective outside one’s own, a notion captured in the concept “perspective taking” assessed within Davis’s (1983) Interpersonal Reactivity Index (IRI).

#### Dimension 4: appraisal/decision (self-focused)

Dimension four may refer to either intention or behavior, and/or any consolidated version of the observed and processed information resulting in a decision or outcome. This dimension is described by Stein (1917) as the “synthesis of experience” whereby the information is processed, and understood, and the individual now is forming a decisive act or intent to respond (or not respond) in a given manner. Broadly, dimension 4 describes the manner and extent to which the observer is impacted by the observed; in research, this domain is commonly assessed through tools like the Empathy Quotient (EQ) a measure most commonly associated with clinical evaluation of Autism (Baron-Cohen and Wheelwright, 2004).

Dimension 4 does not necessarily need to manifest in a behavior, it may lead to behavior, as intention is widely understood as an immediate antecedent of behavior, as described in the theory of planned behavior (Ajzen, 1991; Ajzen, 2012). It is also important to remember that the nature of the response, is not directly implied by this domain (Eisenberg and Miller, 1987). In other words, regardless of whether a response is prosocial, neutral or antisocial, dimension 4 refers to the cumulative outcome of having moved through the previous stages of empathy processing, and any resultant synthesis or conclusive understanding of the event.

### A neural model for the transdisciplinary framework for empathy research

This section will discuss proposed underlying neural mechanisms in terms of structural and functional connectivity across subcortical and cortical regions. Although the neural model proposed here is presented in a linear manner, appearing to begin at the retina and end in the prefrontal cortex, the actual nature of functional connections between structures is multidirectional and likely represents many concurrent, cyclical interactions (Cruz et al., 2023). It is important to emphasize that the aim of this model is functional. The intent is to provide a unified framework and shared lexicon that can be readily applied across disciplines to promote greater consistency and synthesis within the empathy literature. Methodological and operational inconsistencies have created significant gaps that limit the translation of empathy research into practical applications. Rather than proposing a new definition of empathy, this model integrates select, commonly employed theories and organizes their constructs sequentially and clearly. The contribution lies in consolidating existing knowledge within a universal structure designed to reduce methodological variability and enhance the translational potential of cross-disciplinary findings.

### Multi-directional processing

The majority of cortical connectivity is assumed to be bilateral; neuronal communication depicted here primarily involves glutaminergic signaling (Blumenfeld, 2010). The majority of connections described below are initiated by the retinogeniculostriate (V1) and divergent retinal pathways, whereby retinal ganglia (parvocellular, magnocellular, koniocellular, intrinsically photosensitive and melanopsin-containing ganglia) running along the optic nerve, send projections to multiple destinations including the lateral geniculate nucleus (i.e., retinogeniculostriate), suprachiasmatic nucleus and olivary pretectal nucleus (retinohypothalamic pathway), and superior colliculus (retinotectal pathway) (Purves et al., 2018).

**Figure 3:**
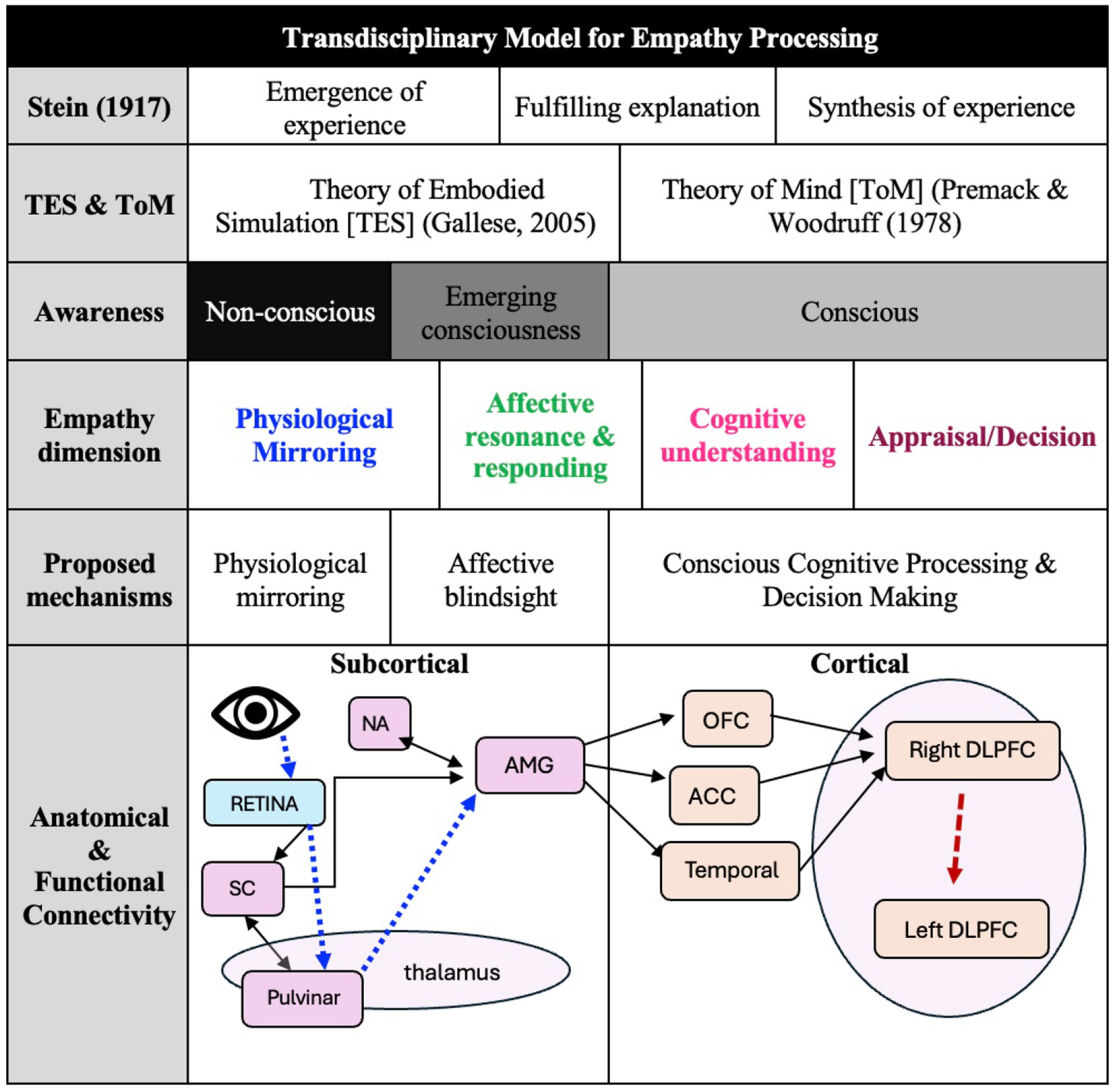
Transdisciplinary framework for empathy research. Historical theories, levels of awareness, empathy dimensions, neural mechanisms, and associated anatomical structures are integrated within a unified conceptual framework. The blue dotted lines represent V1, standard visual pathway; the red dotted line represents an inhibitory connection extending from the right to the left dorsolateral prefrontal cortex.

The primary focus for empathy processing is on the tertiary visual (i.e., retinotectal) pathway, in which retinal ganglia project into the superficial layers of the superior colliculus (Purves et al., 2018). Broadly, these retinal ganglia send glutaminergic inputs to ionotropic AMPA & NMDA receptors as well as some metabotropic glutamate receptors (Binns and Salt, 1994); these projections relay excitatory signals to collicular relay neurons and inhibitory interneurons, as well, which propel through subcortical regions, including the amygdala, toward various cortical areas (Koller et al., 2019). The transition from subcortical to cortical is conceptualized here as marking the gradual transition from non-conscious (subcortical) to conscious (cortical) processing. Figure 3 presents an integrated conceptual model, displayed concurrently with the proposed neural model.

Traditional assumptions of conscious processing as being primarily “top-down,” such that cortical signals are directed downward for implementation via subcortical structures has recently been brough under question (Cruz et al., 2023). Instead, newer technologies have given rise to methods allowing researchers to gain more detailed insight into structural and functional connections across the brain, and modern theorists, including Cruz et al. (2023) propose a more collaborative, multi-directional process, where subcortical signaling and perceptions may influence cortical processes, and vice-versa. This collaborative model of neural processing as neither top-down, nor bottom up – but dynamic and multi-directional, is in direct alignment with the transdisciplinary model for empathy research, proposed here.

#### Dimensions 1 and 2.1: non-conscious mirroring

This stage is characterized by the non-conscious, involuntary mirroring responses described in dimensions 1 and 2, which are consistent with the Theory of Embodied Simulation (TES) (Gallese, 2005) and Stein’s (1917) first stage, described as the “emergence of experience” whereby there is a sudden, urgent and non-descript sense of awareness concerning something or someone observed. In humans, the physiological mirroring network (parietofrontal mirror system) involves the parietal lobe, premotor cortex and the caudal domain of the inferior frontal gyrus (Cattaneo and Rizzolatti, 2009; Kilner et al., 2009). The parietofrontal mirroring system emulates observed voluntary movements (Cattaneo and Rizzolatti, 2009), however, the process of neural simulation of this movement in the observer, tends to be automatic, immediate, may not be consciously experienced. Mirroring process can be somatosensory, physical responses, or they can be affective responses; both represent non-conscious, instantaneous replication or modeling of an observed movement or affect; they are extremely rapid, and may involve actual pre-motor or motor responses, with or without corresponding muscle activation (Schmidt et al., 2021; Simon and Gutsell, 2021). ERP studies also support temporal sequencing of both physiological (mirroring) and affective processes via subcortical structures (Meconi et al., 2018) The defining characteristic of dimensions 1 (physiological mirroring) and 2.1 (affective resonance), is that these experiences are a non-conscious, immediate, physiological or emotional replication of an observed circumstance.

##### Affective blindsight

This early phase of empathy processing involves subcortical pathways typically associated with visual perception, including forms of blindsight (Henley, 2020; Purves et al., 2018), or in particular, affective blindsight, which is the non-conscious processing of social and affective information through the visual system (de Gelder et al., 2005; de Gelder and Tamietto, 2010). It is important to note, the emphasis here is not on visual sensory perception (V1 primary visual pathway), but instead is on secondary pathways, that bypass V1. Affective blindsight phenomena (e.g., perception of social and emotional information without vision) continue to occur even in populations with complete V1 blindness.

Some forms of social and affective information can be perceived through secondary visual pathways, not association with the traditional sensory process that is “vision”. Instead, in cases of affective blindsight, social and emotional information is perceived and processed non-consciously through subcortical pathways, bypassing the primary visual cortex; this phenomena occurs within all mammals, with or without traditional vison intact (de Gelder et al., 2005; de Gelder and Tamietto, 2010). Tamietto and de Gelder (2010) proposed a neural model underlying affective blindsight processing, which involves a subset of retinal ganglia that project to the pulvinar and superior colliculus, looping through adjacent subcortical structures including the amygdala, and related limbic structures responsible for immediate, non-conscious processing of emotional signals (pp. 700). Recent studies involving diffusion tractography have supported the structural and functional connectivity support the model of connectivity presented originally by Tamietto and de Gelder (2010) as underlying blindsight processes; specifically, the connectivity between the retina to the amygdala, via the superior colliculus and pulvinar (Koller et al., 2019).

#### Dimension 2.2: affective response

While dimension 2.1 (affective resonance) refers to the innate, involuntary mirroring of an observed affective state, dimension 2.2, affective responding, refers to one’s own emotional reaction to what has observed. This transition represents an emerging conscious awareness, and marks a critical “self-other” distinction, whereby the mirroring ends, and the person forms their own response to the circumstance observed. In Stein’s (1917) model, dimension 2.2 (affective responding) corresponds the end of the “emergence of experience” and the beginning phase of “fulfilling explanation.” Similarly, this dimension marks the end of phenomena described by Theory of Embodied Simulation (TES) and the transition into phenomena described by the Theory of Mind (ToM). While all dimensions described in this framework are non-linear (e.g., may occur concurrently, or circle back through one another several times) the transition between the first portion of dimension 2 (affective resonance) and the end (affective responding) has a requisite cyclical quality. Specifically, the self-other distinction requires some cycling through dimension 3 (cognitive) in order to achieve the required “self-other” distinction that marks the transition from mirroring what is observed, to the cultivation of a unique emotional reaction to the observed circumstance.

From a structural and functional perspective, this is where the amygdala integrates subcortical input and projects to multiple cortical regions, serving as a bridge between non-conscious and conscious processing systems, akin to the multi-directional conscious and non-conscious processing described by Cruz et al. (2023). The transdisciplinary model for empathy proposed above depicts functional connectivity between the amygdala and key cortical regions, including the orbitofrontal cortex (OFC), anterior cingulate cortex (ACC), and temporal lobe, all of which are well-established in the literature (Blumenfeld, 2010; Cruz et al., 2023). These connections may represent initial awareness of stimuli, which likely evolve to represent more integrative and complex cognitive and emotional processes, as it moves down the ventral stream.

#### Dimensions 3 and 4: conscious cortical processing

This final stage represents the transition to conscious, higher-order processing, involving executive and regulatory cortical regions. This is the final step in the proposed “empathy as a perceptual process” model. Activities in dimensions 3 and 4 are conscious, corresponding with higher order cognitive processing (e.g., perspective taking, as described by Davis, 1983) and decisions that establish one’s intent for responding to what has been observed, corresponding with the Theory of Mind. Stein (1917) describes this phase as “synthesis of experience” whereby the observer makes sense of what has been observed and decides what to do about it. The Decety and Jackson (2006) model invoke several regions in association with empathy processes including the insula, anterior cingulate cortex, and right temporoparietal regions. Indeed, there is a structural and functional basis to this claim, as these activities may be supported by projections from the amygdala to the anterior cingulate cortex (ACC), orbitofrontal cortex (OFC) and temporal regions, which converge functionally with the dorsolateral prefrontal cortex (DLPFC) (Blumenfeld, 2010). Similarly, Meconi et al. (2018) suggests cognitive stages (e.g., mentalizing) of empathy occurs after mirroring and affective processes and involve prefrontal and temporal cortices.

##### Dorsolateral prefrontal cortex (DLPFC)

**Figure 4:**
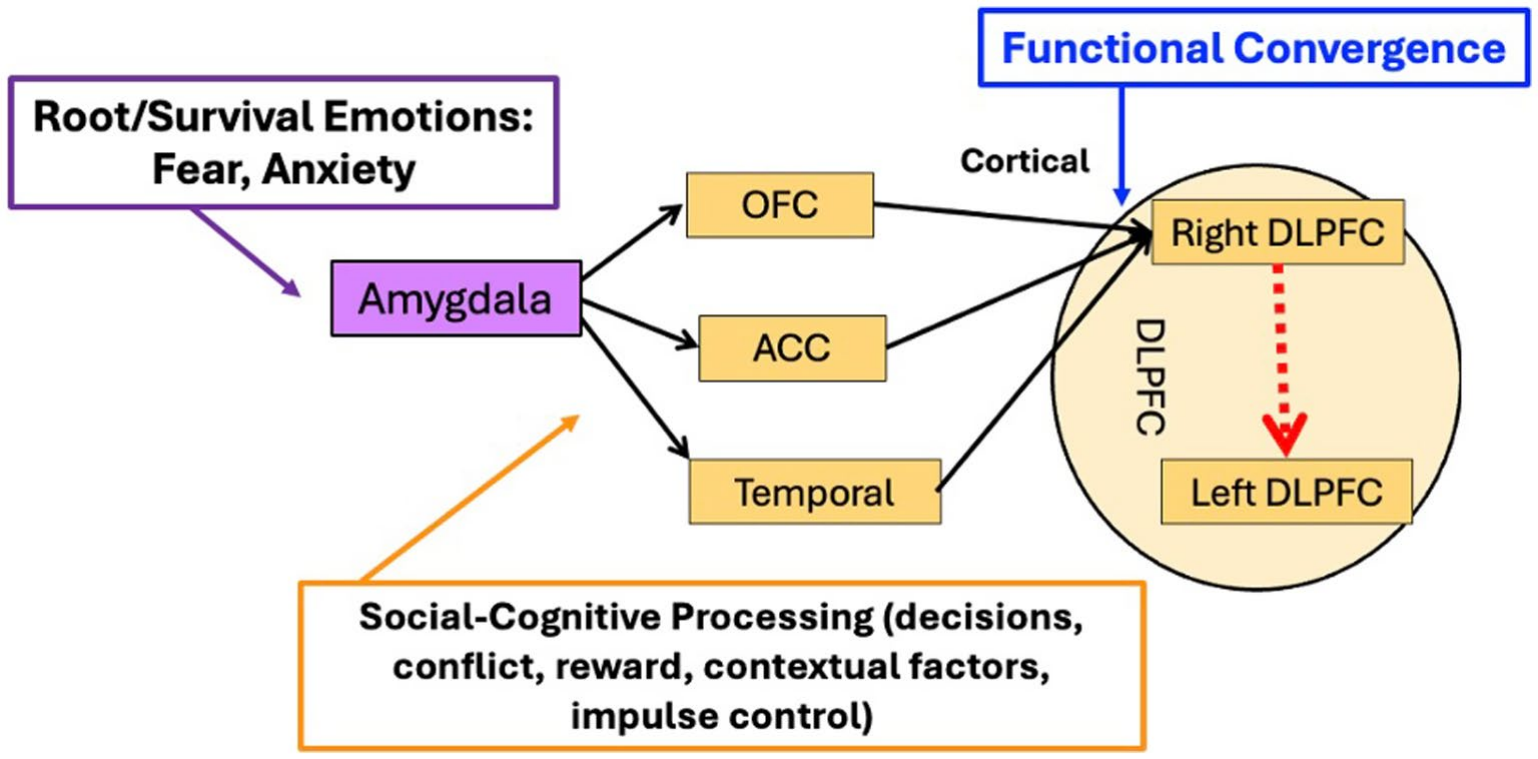
Proposed neural pathways associated with empathy. Schematic illustrating relationships among neural regions implicated in empathy, including the amygdala, orbitofrontal cortex (OFC), anterior cingulate cortex (ACC), temporal cortex, and dorsolateral prefrontal cortex (DLPFC), from affective processing through social-cognitive appraisal and decision-making.

The dorsolateral prefrontal cortex (DLPFC) is a key region involved in the regulation of complex, future-oriented, and flexible human behaviors (Blumenfeld, 2010; Purves et al., 2018). Anatomically, it includes Brodmann areas 9 and 46 and maintains extensive connections with the orbitofrontal cortex, anterior cingulate cortex, premotor cortex, and parietal regions associated with attention and cognitive control (Purves et al., 2018). Functionally, the DLPFC operates as a regulatory hub, integrating input and directing output in a way that shapes decision-making and goal-directed behavior (Purves et al., 2018). It plays a crucial role in short-term memory and the retention of abstract rules, which guide adaptive behavioral responses across changing contexts (Purves et al., 2018) (see Figure 4).

##### Functional connectivity of the dorsolateral prefrontal cortex

The dorsolateral prefrontal cortex (DLPFC) plays a central role in executive function, cognitive control, and emotion regulation, and is specifically associated with response inhibition and emotional and social reactivity (Rêgo et al., 2015; Sergiou et al., 2020). These functions are facilitated by a complex network of functional and structural connections with other cortical and subcortical regions (Blumenfeld, 2010). Understanding the DLPFC’s connectivity provides critical insight into its role in processes such as empathy, social decision-making, and modulation of affective states.

Functionally, the DLPFC maintains bidirectional connections with several key regions, including the orbitofrontal cortex (OFC), anterior cingulate cortex (ACC), and temporal lobes (Blumenfeld, 2010). These connections are not strictly hierarchical but operate dynamically within large-scale neural networks, often described along dorsal and ventral streams of processing (Blumenfeld, 2010; Purves et al., 2018). Each stream contributes uniquely to the integration of cognitive and affective information. The OFC, involved in outcome valuation, affective salience, and reward-based decision-making, shares strong ventral stream connectivity with the DLPFC (Rosenbloom et al., 2012). These connections may facilitate integration between emotional and cognitive domains, supporting socially adaptive behavior and regulation of goal-directed responses. The ACC also exhibits robust connectivity with the DLPFC (Rosenbloom et al., 2012). The dorsal ACC (dACC), associated with cognitive conflict monitoring and attention, aligns more closely with dorsal stream processing (Ebitz et al., 2020). In contrast, the rostral and ventral ACC (rACC/vACC) are more strongly implicated in affective processing and are functionally coupled with the ventral stream (Ebitz et al., 2020). The ACC’s integrative role may enable the DLPFC to evaluate conflicting emotional and cognitive inputs and exert regulatory control over emotional responses. Additionally, the DLPFC maintains functional connections with the temporal cortex, particularly the superior temporal sulcus, anterior temporal pole, and medial temporal structures, all of which are involved in social perception, language, and working memory (Holt et al., 2008). These projections, largely via ventral stream pathways such as the uncinate fasciculus, enable the DLPFC to incorporate socially and emotionally relevant context into regulatory decision-making (Holt et al., 2008).

##### Hemispheric asymmetry in the dorsolateral prefrontal cortices

Inhibitory projections extend from the right DLPFC to the left, indicating lateralization of regulatory control (Blumenfeld, 2010). These prefrontal regions are involved in cognitive appraisal, decision-making, inhibition, and regulation of emotional responses, integrating both affective and cognitive inputs (Blumenfeld, 2010; Purves et al., 2018). Several studies support asymmetric processing (i.e., lateralization) of the right and left dorsolateral prefrontal cortices, and their distinct roles in emotional processing (Cao et al., 2018; D’Alfonso et al., 2000; George et al., 1996; Knoch et al., 2006a; Pascual-Leone et al., 1996; Wyczesany et al., 2022). Broadly, the right DLPFC tends to be associated more with attention to negative emotional states, whereas the left attends more to positive states (Purves et al., 2018). Lesion studies support this as well; symptoms characteristic of right dorsolateral prefrontal lesions includes increased negative affect, whilst persons with lesions on the right are characterized as “unduly cheerful” (Purves et al., 2018, p.716).

Evidence from direct-brain stimulation studies also support the lateralization of the right and left DLPFC (Cao et al., 2018; D’Alfonso et al., 2000; George et al., 1996; Knoch et al., 2006a; Knoch et al., 2006b; Pascual-Leone et al., 1996; Verstraelen et al., 2020; Wyczesany et al., 2022). The theory being that the right DLPFC collects important social and emotional information from numerous inputs, including from the ACC, OFC and temporal regions, consolidates it, and pass this along via inhibitory projections to the left DLPFC, where appraisals and decisions are made. This premise of lateralized function in the dorsolateral prefrontal cortex comprises the foundation of clinical intervention studies using disinhibition of the right DLPFC via low-frequency transcranial magnetic stimulation (1 Hz) in effort to produce antidepressant effects (Bermpohl et al., 2006; Grimm et al., 2008; Lee and Kim, 2018; Luber and Lisanby, 2014; Pallanti et al., 2012). Similarly, other studies have found antidepressant effects via the application of high-frequency (>1 Hz) stimulation to the left DLPFC directly (Asl and Vaghef, 2022; George et al., 1996; Light et al., 2019; Pascual-Leone et al., 1996). Both clinical interventions infer a lateralized, inhibitory connection between the right and left DLPFC, either by disinhibiting the inhibitory connection between right and left or simply applying direct stimulation to the left DLPFC alone.

### Summary and review of the model

Early phases of empathy processing characterized by automatic, non-conscious mirroring physiological mirroring (dimension 1) and affective resonance & responding (dimension 2) are captured by the Theory of Embodied Simulation (TES) and Stein’s first stage of empathy (“emergence of the experience”). This paper proposes that these functions are initiated via the visual systems, including non-image producing perceptual functions, and are mainly facilitated by the secondary and tertiary visual pathways, the same implicated in blindsight phenomena (Tamietto and de Gelder, 2010). In other words, visual facilities in their standard sense are unnecessary, as one may be unable to see entirely, and still process incoming information from within the visual field through non-primary, non-image producing visual pathways.

In research, dimension 1 (physiological processing) and dimension 2 (affective response and responding) are largely measured through physiological tools, such as EMG, pupillary changes, and/or by changes in skin conductance, given that responses are involuntary and occurring outside one’s immediate awareness. Other tools may include indirect measures like fMRI, whereby a BOLD signal in a given location is interpreted in association with a specific emotional response, such as fear. Dimension 2.2 (affective responding) is a bit different, as this stage represents an emerging awareness of one’s own emotional reaction to an external circumstance, and as such, could simply be posed as a question referencing what the person, themselves, is feeling in response to the stimulus.

Later stages of empathy processing (dimension 3) cognitive understanding and dimension 4 (appraisal/decision) are more closely aligned with the Theory of Mind, and Stein’s later phases of empathy, including “fulfilling explanation” which aligns with dimension 3 (cognitive understanding), and “synthesis of experience”, which aligns with dimension 4 (appraisal/decision). Dimensions 3 and 4 reflect deliberate, conscious efforts whereby social, affective and related environmental information has reached conscious awareness, and is reconciled from within one’s personal perspective. Dimension 3 begins with a differentiation of self and other, and it may include a variety of mental exercises such as perspective taking as described by Davis (1983), identifying the emotional state of the other, or it may even consist of an ongoing reflective process, one that persists even after the precipitating event has concluded. Dimension 4 (appraisal/decision) is the outcome of having synthesized information from dimension 3 and is now contending with an evaluation of what to do or not do in response. Dimension 4 may involve a specific behavioral response, a subjective intention, or it may reflect passive disengagement, whereby the person simply ceases to think about the event, all together.

Dimensions 3 and 4 involve higher-order cognitive functions primarily involving the anterior cingulate cortex, orbitofrontal cortex, and temporal regions projecting to the right and left dorsolateral prefrontal cortex. Collectively, the DLPFC can be considered a hub for coordinating high-level, conscious cognitive appraisal with immediate, less-conscious affective processing. This functional integration is particularly relevant in the context of empathy, social judgment, and ideological processing, where regulation of emotionally salient stimuli often interacts with abstract reasoning, personal values, and interpersonal dynamics. The defining aspects of dimensions 3 and 4 are that they are conscious, deliberate and involve an acute awareness of the observer as separate from the observed.

In research, dimension 3 (cognitive understanding) is often measured via perspective taking tasks, whereby a person imagines themselves as the other person, or is tasked with identifying the emotional state, motives or thoughts of the other person, along with other contextual factors, or, in standardized measures such as the Interpersonal Reactivity Index (Davis, 1983), items within the subscale of “perspective taking” are another example of how this domain is assessed. In most empathy research, dimension 4 (appraisal/decision) are typically assessed in terms of valence, such as prosocial, neutral or antisocial responses in a simulated event, and/or in terms of behavioral outcomes (i.e., decision to engage or disengage). In standardized measures, intention may be assessed directly through items inquiring about planned behavior, such as in the Social Justice Scale (Torres-Harding et al., 2012), or in other items asking how a person would respond in response to a given event.

## Limitations

The theory proposed here is a limited by several factors, including the multidisciplinary scope of the literature reviewed. This paper primarily focused on select social sciences and neuroscience research and theories, consolidating differences in methodological approaches and conceptual frames employed to study empathy. Contributions of other more comprehensive empathy research, including concepts and measures from other disciplines would further build out this model. Even in its current state, this paper is additionally limited by scope; a comprehensive philosophical and epistemological analysis from behavioral, psychological and other related perspectives is simply not feasible to address within this introductory model. Yet, it would be greatly advantageous for future research to build out this model further, starting with empathy as a neutral perceptual process, and ultimately extending into related behavioral outcomes and their interpersonal implications. Such work may make a tremendous contribution to both philosophical and clinical research. Further, the author acknowledges that by proposing yet another, new framework to conceptualize empathy, there is an inherent risk of compounding the very problem this paper intends to address.

Further, the neural mechanisms proposed as faciliatory of the above empathy dimensions often rely on indirect measures such as fMRI (Decety and Jackson, 2006); to establish causal relationships direct measures of brain activity are necessary. However, at present most direct measures are not feasible or safe for non-invasive human research due to the involvement of subcortical structures.

Additionally, the present theory addresses processes occurring mainly through structural and functional connections within the larger visual system. Although traditional vision is not necessary for blindsight perceptions, this model does prioritize contributions made through secondary visual pathways and does not comprehensively address contributions made through many other sensory systems, such as auditory or tactile. Future works may wish to expand this model to incorporate contributions made via other sensory systems. A special note can be made concerning non-visual, internal representations such as those arising when reading, and other similar phenomena captured in Davis’ (1983) index; although non-visual stimuli may generate empathic responses, the process may involve differences across dimensions 1 and 2, which address physiological and affective mirroring, due to the absence of visual stimuli. Additional research is needed to further explore this domain.

Lastly, and perhaps most importantly, the utility of this model is directly dependent on the extent to which it is employed or its lexicon used by other empathy researchers, either directly, simply by using this shared lexicon when discussing empathy, or retrospectively, in translational efforts to consolidate multiple empathy studies, that extend across disciplines.

## Conclusion

Empathy research has long been fraught with conceptual inconsistencies. Divergent theories and incompatible methods have produced fragmented findings that hinder synthesis and translational application of findings, echoing the problems Edith Stein identified over a century ago. Empathy research has tremendous potential to address a wide array of social and interpersonal problems. Yet, a cohesive conceptual framework is necessary to allow findings from multiple disciplines, conceptual orientations and methods to be synthesized coherently, and ultimately, translated into real-life applications. This paper presents a consolidated model for empathy research that intends to do just this; by reframing empathy as a neutral perceptual process, within a unified transdisciplinary framework findings from multiple academic domains can be understood as different corners of the same conceptual whole. This model is functional rather than comprehensive; its novel contribution lies in synthesizing existing empathy theories into a unified, cross-disciplinary framework and shared lexicon to improve consistency and translational utility in empathy research.

This model does carry important social implications. By defining empathy in more pragmatic, neutral terms, it reduces the risk of the concept being distorted or misused as a proxy for moral value. Reframing empathy as a perceptual phenomenon also makes the concept more accessible to scientific inquiry. Drawing from Stein’s (1917) conceptualization of empathy as an experience *sui generis*, the model presented here integrates two of the most predominantly referenced empathy theories, Theory of Mind (ToM) and Theory of Embodied Simulation (TES), within a four-stage structure encompassing (1) physiological mirroring, (2) affective resonance and responding, (3) cognitive and (4) appraisal/decision. By linking these united conceptual domains to their neural underpinnings, this testable framework provides both theoretical unity and a lexicon accessible across disciplines, which may help researchers synthesize their work, in order to find new strategies to counteract dehumanization, improve diplomacy, foster human connection and enhance quality of life, overall.

## Data Availability

The original contributions presented in the study are included in the article/supplementary material, further inquiries can be directed to the corresponding author.
